# Signal mining of adverse events of proteasome inhibitors in multiple myeloma based on FAERS

**DOI:** 10.3389/fphar.2024.1396378

**Published:** 2024-09-03

**Authors:** Yuan Peng, Yuying Zhou, Kaisen Shu, Xu Jia, Yan Zhong

**Affiliations:** ^1^ Department of Pharmacy, Affiliated Hospital of North Sichuan Medical College, Nanchong, Sichuan, China; ^2^ School of Pharmacy, North Sichuan Medical College, Nanchong, Sichuan, China; ^3^ The First Clinical Medical College, Xuzhou Medical University, Xuzhou, Jiangsu, China; ^4^ Department of Pharmacy, Hospital of Chengdu Office of People’s Government of Tibetan Autonomous Region (Hospital.C.T), Chengdu, Sichuan, China

**Keywords:** proteasome inhibitors, adverse event, signal mining, multiple myeloma, adverse event reporting system

## Abstract

**Objective:**

To mine and analyze adverse events (AEs) related to proteasome inhibitors in multiple myeloma based on the FDA Adverse Event Reporting System (FAERS), providing references for rational clinical medication.

**Methods:**

AE data related to multiple myeloma proteasome inhibitors were collected from the FAERS from the first quarter of 2010 to the first quarter of 2024. Signal mining of AEs was conducted using the reporting odds ratio method and Bayesian confidence propagation neural network method.

**Results:**

A total of 8,805 reports for bortezomib, 5,264 for carfilzomib, and 8,771 for ixazomib were collected, with corresponding AE signals of 474, 279, and 287, respectively, involving 23, 21, and 22 System Organ Classes (SOCs). The report information for the three drugs tended to be consistent: more cases were reported in males than in females; the majority of patients were 65 years and over; AEs mostly occurred within 6 months of medication; the outcomes primarily consisted of hospitalization, prolonged hospital stay, and other serious adverse events; the primary reporting country was the United States. The most affected SOCs were infections and infestations, general disorders and administration site conditions, and blood and lymphatic system disorders.

**Conclusion:**

The overall distribution of AEs for the three multiple myeloma proteasome inhibitors was consistent, but there were certain differences in specific AE signal characteristics, which should be noted in clinical applications.

## 1 Introduction

Multiple myeloma (MM) is the second most common malignant hematologic tumor, characterized by the proliferation of abnormal plasma cells producing monoclonal immunoglobulins, leading to symptoms such as bone pain, anemia, renal impairment, and hypercalcemia (Landgren, 2013; Kazandjian, 2016). It is more common in middle-aged and elderly individuals, and its incidence is rising with the increasing aging population in China (Kyle et al., 2003; Liu et al., 2019). For MM patients, traditional chemotherapy often results in complete remission rate and short median survival (Fu et al., 2013). However, the emergence of new anticancer drugs, proteasome inhibitors (PIs) (Perea et al., 2017), which inhibit the function of tumor proteasomes, disrupt the proliferation and differentiation of tumor cells, and accelerate their collapse and apoptosis, has improved the remission rate and survival period for patients (Rajkumar et al., 2005; Pakjoo et al., 2024). The currently available PIs for multiple myeloma include bortezomib, carfilzomib and ixazomib (Stewart et al., 2015; Ettari et al., 2016). In 2003, bortezomib was approved as the first-generation PI. In 2012, carfilzomib was approved in the United States for patients with MM who had received at least two prior therapies and had relapsed. In 2016, carfilzomib received further approval from the United States for use as a monotherapy or in combination therapy for patients with relapsed or refractory MM. Ixazomib, the world’s first oral PI, was approved for marketing in the United States in 2015. It is commonly used in combination with lenalidomide and dexamethasone for adult MM patients who have received at least one prior therapy. Over the past two decades, PIs have become an essential medication for treating MM patients. Bortezomib has been recommended as a first-line treatment for MM in guidelines in both the US and China, and carfilzomib and ixazomib have also been included in first-line treatment plans (Luo et al., 2018; Witteles and Liedtke, 2019). Therefore, understanding the safety of PIs is of significant importance for their rational clinical use.

The FDA Adverse Event Reporting System (FAERS) is a database established by the FDA to support post-market safety monitoring studies for drugs (DeLoughery and Shatzel, 2019). It includes reports submitted by consumers, healthcare professionals, and drug manufacturers from 1968 to the present. The FDA releases the data to the public every quarter for free download. It offers a broad detection scope and is not limited by time or geography (Huang et al., 2021). The database includes seven data sets, which are linked through the “primaryid” or “ISR” primary link field. Due to its large volume of data, the wealth of information, and free access to the public, this study aims to analyze the adverse event (AE) data of PIs through FAERS to identify AE signals of PIs and to provide references for the rational use of these drugs (Mina et al., 2021).

## 2 Materials and methods

### 2.1 Data source

This study collected AE data from FAERS covering 57 quarters from the first quarter of 2010 to the first quarter of 2024, including patient personal information records, drug use records, AE records, drug treatment duration, and AE outcomes.

### 2.2 Data processing

Bortezomib was searched under the names “Bortezomib,” “Velcae,” “LDP341,” “PS341”; carfilzomib as “Carfilzomib,” “Kyprolis,” “PR171”; and ixazomib as “Ixazomib,” “Ninlaro,” “MLN 9708” to obtain primary suspect (PS) drug reports. FAERS uses the preferred terms (PTs) from the Medical Dictionary for Regulatory Activities (MedDRA) to code AEs (Taher et al., 2021).

### 2.3 Mining method

This study used the Reporting Odds Ratio (ROR) method and the Bayesian Confidence Propagation Neural Network (BCPNN) method to mine the data. Calculations for both methods are based on a fourfold table of disproportionality measures, as shown in Table 1. The ROR method has the advantage of eliminating bias and offering high sensitivity, but it is prone to false positives. The BCPNN method, which adds Bayesian law to the fourfold table, makes the results more stable and specific (Ma et al., 2021; Zou et al., 2023). This study used these two methods to investigate the same signals mined, thereby reducing bias caused by a single algorithm, and the algorithms of the specific methods are shown in Table 2.

**TABLE 1:** Four-cell table of proportional imbalance method.

Project	Target AEs	Other AEs	Total
Target Drug	a	b	a + b
Other Drugs	c	d	c + d
Total	a + c	b + d	N = a + b + c + d

Note: a, the number of reports with suspect adverse event (AE) of the suspect drug; b: the number of reports with the suspect AE, of all other drugs; c: the number of reports with all other AEs, of the suspect drug; d: the number of reports with all other AEs, of all other drugs.

**TABLE 2:** Signal detection calculation method.

Method	Calculation formula	Judgement criteria
ROR Method	$\text{ROR}=\frac{a*d}{b*c}$	95%CI > 1, a ≥ 3
	$95\%\text{CI}=e^{\ln ROR1.96-\sqrt{\frac{1}{a}+\frac{1}{b}+\frac{1}{c}+\frac{1}{d}}}$	
BCPNN Method	$\text{IC}=\log_{2}\frac{a\left(a+b+c+d\right)}{\left(a+b\right)\left(a+c\right)}$	95%CI > 0, a ≥ 3
	$\gamma =\gamma_{11}\frac{\left(N+\alpha \right)\left(N+\beta \right)}{\left(a+b+\alpha_{i}\right)\left(a+c+\beta_{1}\right)}$	
	$E\left(\text{IC}\right)=\log_{2}\frac{\left(a+\gamma_{11}\right)\left(N+\alpha \right)\left(N+\beta \right)}{\left(N+\gamma \right)\left(a+b+\alpha_{i}\right)\left(a+c+\beta_{1}\right)}$	
	$V\left(\text{IC}\right)={\left(\frac{1}{\log2}\right)}^{2}\left[\frac{N-a+\gamma -\gamma_{11}}{\left(a+\gamma_{11}\right)\left(1+N+\gamma \right)}+\frac{N-a-b+\alpha -\alpha_{i}}{\left(a+b+\alpha_{i}\right)\left(1+N+a\right)}+\frac{N-a-c+\beta -\beta_{1}}{\left(a+c+\beta_{1}\right)\left(1+N+\beta \right)}\right]$	
	$\text{SD}=\sqrt{V\left(IC\right)}$	
	$95\%\text{CI}=E\left(\text{IC}\right)-2\text{SD}$	
	$\alpha =\beta =2,\;\alpha_{1}=\beta_{1}=1,\;\gamma_{11}=1\;N=a+b+c+d$	

Note: ROR, reporting odds ratio; BCPNN: bayesian confidence propagation neural network; CI: confidence interval; 95%CI: the lower limit of the 95% confidence interval.

### 2.4 Signal categorization

The signals identified were organized using the System Organ Class (SOC) (Liu et al., 2019) of the MedDRA, version 23.1, in English.

## 3 Results

### 3.1 Basic information on AE reports

A total of 22,840 AE reports were collected, involving patients with PIs as the PS. Of these, 8,805 were for bortezomib, 5,264 for carfilzomib, and 8,771 for ixazomib. The reports for all three drugs shared the following characteristics: there were more cases in males than females; the main age group was centered around 65 years and older; AEs predominantly occurred within 6 months of treatment; and outcomes primarily involved hospitalization, prolonged hospital stays, and other serious adverse events; the United States was the primary reporting country. Specific information is shown in Table 3.

**TABLE 3:** Basic information of AE reports concerned patients.

Category		Bortezomib		Carfilzomib		Ixazomib	
			Reported number		Reported number		Reported number
Total		8805		5264		8771	
Gender	Female		2490		2197		4018
	Male		3466		2878		4622
	Missing		2849		189		131
Age/year	<18		196		63		27
	18∼39		106		56		27
	40∼64		2092		2030		1579
	≥65		3147		2669		4555
	Missing		3264		446		2583
Duration of medication/d	≤7		1105		744		874
	8∼30		1366		820		867
	31∼180		1978		1182		1411
	181∼365		445		398		514
	>365		493		349		683
	Missing		3418		1771		4422
Outcome	Death		1244		623		1663
	Disability		180		82		33
	Hospitalization—initial or prolonged		3355		2463		3202
	Life-Threatening		305		262		70
	Required intervention to prevent permanent impairment/damage		7		3		5
	Congenital anomaly		0		1		1
	Other serious		3714		1830		3797
Occr_country		America	1759	America	1422	America	3466
		France	1053	France	481	Canada	1628
		Germany	459	Japan	475	Japan	1120
		Japan	454	Germany	385	Germany	628
		Spain	400	Britain	306	France	421

Note: Outcome, the patient outcomes for the event; Occr_country, the country where the event occurred (top 5 reported countries).

### 3.2 AE signals analysis

#### 3.2.1 SOCs distribution

**FIGURE 1:**
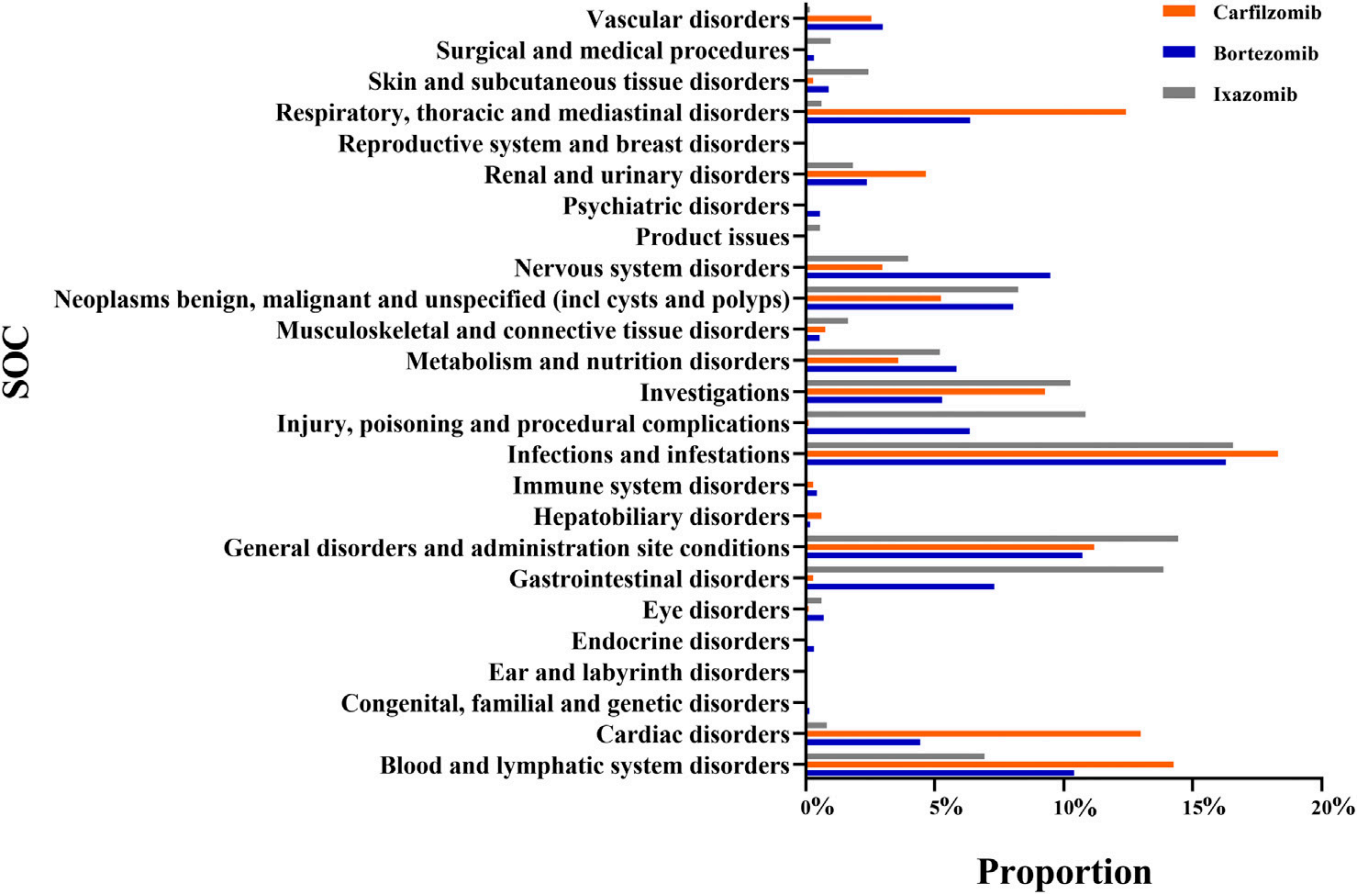
Proportion of Proteasome Inhibitor-related AEs at the SOC level.

The number of AE signals identified by the ROR method was 714 for bortezomib, 493 for carfilzomib, and 474 for ixazomib. The number of AE signals identified by the BCPNN method was 474 for bortezomib, 279 for carfilzomib, and 287 for ixazomib. A total of 1,040 signals were identified for PIs by both methods in common, with the number and proportion of AE signals for bortezomib, carfilzomib, and ixazomib being 474 (45.58%), 279 (26.83%), and 287 (27.59%), respectively. The number of affected SOC categories was 23 for bortezomib, 21 for carfilzomib, and 22 for ixazomib. The main SOCs were infections and infestations, general disorders and administration site conditions, and blood and lymphatic system disorders. Additionally, bortezomib was associated with nervous system disorders (9.49%), respiratory, thoracic, and mediastinal disorders (6.36%), and gastrointestinal disorders (7.30%). Carfilzomib was associated with cardiac disorders (12.98%), respiratory, thoracic, and mediastinal disorders (12.41%), and others. Ixazomib was associated with gastrointestinal disorders (13.87%), investigations (10.25%), etc. See Figure 1 for details.

#### 3.2.2 Siganls distribution

In the top 20 AE reports for bortezomib, carfilzomib, and ixazomib, signals related to blood and lymphatic system disorders were consistently present, including thrombocytopenia and anemia. Additionally, signals such as atrial fibrillation, pneumonia, and sepsis were observed with bortezomib. Carfilzomib was associated with signals like acute kidney injury, cardiac failure, and respiratory failure. Ixazomib showed signals for fatigue, rash, and neuropathy peripheral. For detailed information, see Tables 4–6.

**TABLE 4:** Top 20 signals of bortezomib AE reporting numbers.

Drug	PT	Number of reports	ROR (95% CI lower limit)	BCPNN (95% CI lower limit)
Bortezomib	Plasma cell myeloma^a^	963	44.09	5.19
	Neuropathy peripheral	676	11.74	3.30
	Off label use^a^	654	1.28	0.13
	Death	620	1.27	0.12
	Pneumonia	600	2.93	1.31
	Diarrhoea	596	1.50	0.35
	Thrombocytopenia	539	8.24	2.78
	Pyrexia	522	2.37	0.99
	Anaemia	381	2.99	1.30
	Sepsis	327	4.63	1.92
	Disease progression^a^	320	4.95	2.01
	Hypotension	315	2.59	1.08
	Platelet count decreased	270	3.71	1.58
	Neutropenia	266	3.16	1.35
	Intentional product use issue^a^	244	3.52	1.49
	Constipation	238	1.70	0.45
	Febrile neutropenia	228	5.52	2.13
	Dehydration	222	2.63	1.07
	Cardiac failure	211	4.12	1.70
	Atrial fibrillation	206	3.27	1.37

Note: PT, preferred terms.

^a^ Indicates not mentioned in the instruction.

**TABLE 5:** Top 20 signals of carfilzomib AE reporting numbers.

Drug	PT	Number of reports	ROR (95% CI lower limit)	BCPNN (95% CI lower limit)
Carfilzomib	Pneumonia	440	3.96	1.55
	Death^a^	398	1.49	0.15
	Plasma cell myeloma^a^	379	30.35	4.46
	Pyrexia	367	3.05	1.17
	Dyspnoea	300	1.54	0.18
	Thrombocytopenia	272	7.44	2.42
	Anaemia	254	3.63	1.38
	Acute kidney injury	243	4.22	1.59
	Platelet count decreased	223	5.65	2.00
	Sepsis	215	5.52	1.97
	Cardiac failure	214	7.82	2.47
	Infection	166	3.11	1.11
	Hypertension	159	2.04	0.50
	Atrial fibrillation	158	4.59	1.66
	Respiratory failure	146	5.98	2.03
	Pancytopenia^a^	140	7.57	2.37
	Neutropenia	130	2.72	0.89
	Disease progression^a^	124	3.32	1.17
	Febrile neutropenia	123	5.27	1.83
	Renal failure	123	2.22	0.59

Note: PT, preferred terms.

^a^ Indicates not mentioned in the instruction.

**TABLE 6:** Top 20 signals of ixazomib AE reporting numbers.

Drug	PT	Number of reports	ROR (95% CI lower limit)	BCPNN (95% CI lower limit)
Ixazomib	Plasma cell myeloma^a^	1946	75.27	5.96
	Off label use^a^	1943	3.20	1.50
	Diarrhoea	1623	3.42	1.59
	Death^a^	1509	2.57	1.18
	Pneumonia	1403	5.69	2.31
	Nausea	914	1.58	0.48
	Fatigue	774	1.22	0.10
	Platelet count decreased	720	8.33	2.84
	Thrombocytopenia	688	8.44	2.86
	Neuropathy peripheral	637	8.71	2.90
	Vomiting	636	1.86	0.69
	Asthenia^a^	534	1.82	0.65
	Pyrexia^a^	472	1.68	0.52
	White blood cell count decreased	471	5.16	2.13
	Constipation	468	2.76	1.23
	Rash	466	1.47	0.33
	Anaemia^a^	423	2.64	1.16
	Product dose omission issue^a^	377	2.11	0.83
	Fall^a^	366	1.30	0.14
	Back pain	317	1.57	0.39

Note: PT, preferred terms.

^a^ Idicates not mentioned in the instruction.

## 4 Discussion

### 4.1 Basic situation of AEs

In this study, the number of AE signals identified was 474 for bortezomib, 279 for carfilzomib, and 287 for ixazomib. The most frequently reported signals for bortezomib included neuropathy peripheral, diarrhoea, pneumonia, and thrombocytopenia; for carfilzomib, they were pneumonia, pyrexia, dyspnoea, and acute kidney injury; and for ixazomib, they included diarrhoea, pneumonia, thrombocytopenia, and neuropathy peripheral. These results were consistent with the drug package inserts and related reports, confirming the reliability of this study (Steele, 2013; Bonnet and Moreau, 2017; Cengiz Seval and Beksac, 2018). In the reports for all three drugs, most patients were over 65 years of age, which may be related to the prevalence of the disease in older patients. The reports were primarily from Europe and America, possibly due to the origin of the database and the higher incidence of the disease in these populations (Huang et al., 2022).

### 4.2 Characteristics of PIs involving SOCs

#### 4.2.1 Infections and infestations

In this category, all three drugs showed signals for pneumonia, respiratory tract infection, and lung infection. The propensity for infections in MM patients may be related to the impact of PIs on the immune system. Studies indicate that PIs can increase the risk of viral infections through mechanisms such as disruption of intracellular antigen presentation, exhaustion of alloreactive T cells, and reduction in cytotoxic T cell responses (Teh et al., 2016). Therefore, infection prevention should be considered during medication.

#### 4.2.2 General disorders and administration site conditions

In this SOC, bortezomib, carfilzomib and ixazomib all had the highest number of death signals. Due to the disease’s prevalence in elderly patients with multiple comorbidities, it is currently uncertain if the deaths are drug-related. Additionally, all three drugs showed a significant number of reports for pyrexia. Pyrexia after bortezomib administration has been associated with pro-inflammatory cytokines secreted by macrophages or fibroblasts (Maruyama et al., 2008). No mechanisms have been reported for the other two drugs, but pyrexia is listed as a common adverse drug reaction (ADR) in the carfilzomib package insert, while ixazomib's package insert does not mention it. Therefore, monitoring body temperature is recommended when using PIs.

#### 4.2.3 Blood and lymphatic system disorders

All three drugs showed signals for thrombocytopenia, anaemia and neutropenia, almost consistent with the ADRs listed in their package inserts. Bortezomib has been reported to regulate the Rho/Rho kinase pathway, a negative regulator of proplatelet formation, thereby leading to thrombocytopenia (Murai et al., 2014). However, since MM itself is a blood system disease with features like thrombocytopenia and anemia, the association with PIs requires further investigation.

#### 4.2.4 Gastrointestinal disorders

Bortezomib and ixazomib showed a higher proportion of signals in this category, mainly for diarrhoea and constipation, consistent with package inserts and clinical trial results. Carfilzomib showed the lowest proportion, with signals only for colitis ischaemic, gastrointestinal amyloidosis, neutropenic colitis, suggesting lower gastrointestinal toxicity compared to bortezomib, aligning with the research by Stansborough RL (Stansborough and Gibson, 2017). Studies have shown that bortezomib can induce time-dependent gastrointestinal damage possibly related to the secretion of pro-inflammatory cytokines. However, the mechanisms of gastrointestinal pathology are complex and diverse, and further exploration is needed to understand the mechanisms of PI-induced gastrointestinal toxicity (Maruyama et al., 2008). With unclear mechanisms of gastrointestinal toxicity from PIs, there are currently no effective preventive measures. In cases of severe gastrointestinal reactions during medication, it is advised to discontinue the current medication or switch to carfilzomib with lower toxicity.

#### 4.2.5 Respiratory, thoracic, and mediastinal disorders

Both bortezomib and carfilzomib showed signals for lung infiltration and acute respiratory distress syndrome, which are listed as ADRs in their package inserts. In such cases, it is advised to discontinue the medication and assess the patient promptly. Ixazomib showed signals for upper respiratory inflammation and respiratory symptoms. Currently, the related toxicity mechanisms are unclear, and preventive and treatment measures are still under research. Patients with respiratory diseases should use these drugs cautiously, weighing the risks and benefits, and if used, their respiratory symptoms should be closely monitored.

#### 4.2.6 Cardiac disorders

All three drugs, bortezomib, carfilzomib, and ixazomib, showed severe signals of cardiac failure, cardiac amyloidosis, and acute coronary syndrome. The signals showed by ixazomib also include cardiac dysfunction, cor pulmonale chronic, degenerative aortic valve disease, left atrial enlargement, and right atrial enlargement, but the package insert for ixazomib only mentions cardiac failure and arrhythmia. Studies suggested that the high sensitivity of cardiomyocytes to damage by bortezomib and carfilzomib may be related to their inhibition of high muscle protein turnover, leading to the induction of apoptosis and myocyte death, and consequently cardiac failure (Hasinoff et al., 2017). The toxicity mechanisms of ixazomib may not have been fully explored due to its shorter time on the market. When using PIs, consider their cardiotoxicity, closely monitor cardiac indicators, and if patients show such symptoms, discontinue the medication and assess them immediately.

#### 4.2.7 Renal and urinary disorders

Of the three drugs, only ixazomib’s package insert does not list signals for acute kidney injury and acute renal failure. Studies have found kidney damage to be associated with an increased risk of early death in MM patients, especially in older patients with more comorbidities (Fan et al., 2021). For bortezomib’s renal toxicity mechanism, it is speculated to increase transcription of pro-apoptotic genes bax and puma, key components of the intrinsic apoptosis pathway, and indirectly affect proteins involved in the extrinsic pathway of cell apoptosis, inducing ischemic renal tubular apoptosis, leading to renal function decline or failure (Huber et al., 2009). No mechanisms have been discussed for ixazomib and carfilzomib. However, carfilzomib’s package insert warns of a greater risk of fatal renal failure in patients with reduced creatinine clearance. When treating patients with PIs, it is advised to monitor renal function regularly, to measure serum creatinine or estimated creatinine clearance, and to take intervention measures promptly if kidney damage or failure occurs.

#### 4.2.8 Nervous system disorders

In this study, all three drugs were associated with multiple nervous system disorders. Studies showed that although bortezomib could not penetrate the blood-brain barrier into the central nervous system, it accumulated in dorsal root ganglia (DRG) causing neurotoxicity and indirectly leading to central nervous system dysfunction, including glial activation, glutamate homeostasis disruption, and inflammation (Yamamoto and Egashira, 2021). No mechanisms have been reported for the other two drugs. However, results from the ENDEAVOR randomized controlled trial showed a significantly lower incidence of grade 2 neuropathy peripheral in the carfilzomib group compared to the bortezomib group (6% vs. 32%) (Goldschmidt et al., 2018). Another study comparing subcutaneous and intravenous bortezomib found better tolerance and lower grade 2 neuropathy peripheral with subcutaneous injection (24% vs. 41%) (Moreau et al., 2011). Consider stopping medication, changing the administration method, or switching treatment plans in cases of severe ADRs in the nervous system during PIs treatment.

## 5 Conclusion

This study conducted mining and analysis of AE signals for PIs through the FAERS database using the ROR method and BCPNN method, and conducted detailed discussions on the SOCs such as infections and infestations, blood and lymphatic system disorders, nervous system disorders, gastrointestinal disorders in conjunction with existing data. The study found that the AE signals of bortezomib, carfilzomib, ixazomib were essentially the same for infections and infestations, blood and lymphatic system disorders, and nervous system disorders, but differed in respiratory, thoracic, and mediastinal diseases. Additionally, carfilzomib had lower gastrointestinal toxicity, while ixazomib presented numerous signals not mentioned in its package insert, such as pyrexia, kidney and urinary system disorders, and cardiac disorders. This study not only provides a reference for the rational clinical use of PIs but also promotes drug vigilance work for PIs.

## Data Availability

The datasets presented in this study can be found in online repositories. The names of the repository/repositories and accession number(s) can be found in the article/supplementary material.

## Abbreviations

ADR, adverse drug reaction; AE, adverse event; BCPNN, Bayesian Confidence Propagation Neural Network; DRG, dorsal root ganglia; FAERS, The FDA Adverse Event Reporting System; MedDRA, Medical Dictionary for Regulatory Activities; MM, multiple myeloma; PI, proteasome inhibitor; PS, primary suspect; PT, preferred term; ROR, Reporting Odds Ratio; SOC, System Organ Class.
